# Health Benefits of Uses and Applications of *Moringa oleifera* in Bakery Products

**DOI:** 10.3390/plants10020318

**Published:** 2021-02-06

**Authors:** Paula García Milla, Rocío Peñalver, Gema Nieto

**Affiliations:** 1 Department of Food Technology, Nutrition and Food Science, Veterinary Faculty, University of Murcia, Regional Campus of International Excellence “Campus Mare Nostrum”, Campus de Espinardo, 30100 Espinardo, Spain; ppaulagm@gmail.com (P.G.M.); rocio.penalver@um.es (R.P.); 2 Molecular Microbiology and Food Research Laboratory, Escuela de Nutrición y Dietética, Facultad de Ciencias para el cuidado de la Salud, Universidad San Sebastián, Santiago 8420524, Chile

**Keywords:** *Moringa*, natural preservatives, bakery products, functional food

## Abstract

*Moringa oleifera* belongs to the Moringaceae family and is the best known of the native *Moringa oleifera* genus. For centuries, it has been used as a system of Ayurvedic and Unani medicine and has a wide range of nutritional and bioactive compounds, including proteins, essential amino acids, carbohydrates, lipids, fibre, vitamins, minerals, phenolic compounds, phytosterols and others. These characteristics allow it to have pharmacological properties, including anti-diabetic, anti-inflammatory, anticarcinogenic, antioxidant, cardioprotective, antimicrobial and hepatoprotective properties. The entire *Moringa oleifera* plant is edible, including its flowers, however, it is not entirely safe, because of compounds that have been found mainly in the root and bark, so the leaf was identified as the safest. *Moringa oleifera* is recognised as an excellent source of phytochemicals, with potential applications in functional and medicinal food preparations due to its nutritional and medicinal properties; many authors have experimented with incorporating it mainly in biscuits, cakes, brownies, meats, juices and sandwiches. The results are fascinating, as the products increase their nutritional value; however, the concentrations cannot be high, as this affects the organoleptic characteristics of the supplemented products. The aim of this study is to review the application of *Moringa oleifera* in bakery products, which will allow the creation of new products that improve their nutritional and functional value.

## 1. Introduction

*Moringa oleifera* is a genus of the fast-growing tropical deciduous plant of the Moringaceae family, with thick, tuberous roots, light green leaves and abundant flowering with elongated, pendulous fruits and seeds [1]. It is a native crop of northern India, although it is found in southwest Asia, southwest and northwest Africa and Madagascar. It has long been a part of traditional horticulture, used mainly for ornamental purposes in cities along the Pacific coast of Mexico [2], as well as plantations in Bolivia, Argentina and elsewhere in the world [3]. It has 13 known species, with *Moringa oleifera*, native to India, being one of the most studied and used for its nutritional, phytochemical and pharmacological properties. According to ayurvedic medicine (traditional and alternative medicine of India) [4], it is attributed properties for the treatment of some diseases, such as asthma, epilepsy, eye and skin diseases, fever and haemorrhoids [5,6,7]. In fact, it is a medicinal plant traditionally known in the approach to malnutrition and other diseases [8].

**Figure 1:**
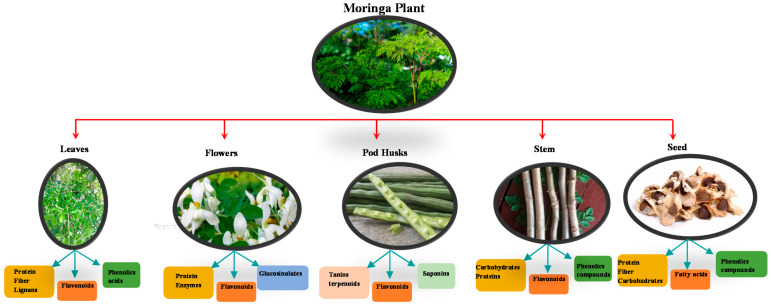
Composition in bioactive compounds of vegetative structures of *Moringa oleifera* plant.

It can withstand long periods of drought, growing well in arid and semi-arid areas. According to researchers, it tolerates soils with a pH between 4.5 and 8, although neutral or slightly acidic pH is more favourable [9]; it is a very adaptable species, lives about 20 years and reaches a height of 5 to 10 m in a short period of time, reaching 4 m in 6 months [10]. It is considered a very versatile plant due to its great capacity to provide edible food, which includes different vegetative structures, such as leaves, pod shells, stem, flowers, fruits and seeds. These structures contain bioactive compounds and nutrients (Figure 1), such as phenolic compounds, fatty acids, carbohydrates, fibre, minerals, vitamins and functional peptides with a wide potential to be used in food.

However, not all of the plant is probably safe, as toxic compounds have been found [11]. On the other hand, its uses are varied, as the seed is used to purify water and the oil from the seeds can be used as a fertiliser [6]. Considering that previous studies have shown that bioactive compounds from herbs and plants could be used for functional food product innovation [11,12,13,14]. *Moringa oleifera* plants could be used for functional food and other industrial food applications [15]. Therefore, *Moringa oleifera* provides nutrients that benefit health, making it a key food for food security in areas with fewer economic resources [16], this review summarises recent knowledge on bioactive compounds from *Moringa oleifera* plants and their potential use in the formulation of food products, especially bakery products. The objective of this review is to know the uses and applications of *Moringa oleifera* in bakery products, in order to know what the quantity or concentration of *Moringa oleifera* is that allows maintaining the sensory characteristics of the product.

## 2. Botanical and Taxonomic Characteristics

*Moringa oleifera* belongs to the Moringaceae family, order Capparidales, class Magnoleopsida. It is the best known of the genus *Moringa oleifera*, which has 13 species [17]. It is identified by its fruit in the form of a long, woody pod, which, when ripe, opens into two leaflets, where it contains the trivalent seeds with longitudinal wings. Its pinnate leaves are divided into leaflets arranged on a rachis [18]. The flowers are grouped in axillary panicles, are bisexual, zygomorphic with five unequal white petals, five sepals, five stamens and several staminodes; they have pedicels and auxiliary inflorescences. The plant has erect stems and tuberous roots [2,19]. It is a tree that can reach 7–12 m in height and 20–40 cm in diameter, with an open crown and straight stem [20].

## 3. Ethnopharmacological Uses of *Moringa oleifera*

*Moringa oleifera* has been used as a medicine in India since the 18th century BC. Traditional healers used different parts of the plant as traditional medicines. The medicinal uses are numerous and have long been recognised as an Ayurvedic and Unani system of medicine. Almost all parts of the plant: root, bark, gum, leaf, fruit (pods), flowers, seeds and seed oil, have been used to treat various diseases, like skin infections, swelling, anaemia, asthma, bronchitis, diarrhoea, headache, joint pain, rheumatism, gout, diarrhoea, heart problems, fevers, digestive disorders, wounds, diabetes, conjunctivitis, haemorrhoids, goitre, earache, measles and smallpox in the indigenous system of medicine [5,21].

## 4. Composition of *Moringa oleifera*

The composition of *Moringa oleifera* varies depending on climatic variations, crop management, whether it is cultivated or wild, the state of maturity of the plant at the time of harvesting, the type of post-harvest processing and depends on the growing area, i.e., the land where it is grown [22].

### 4.1. Primary Metabolites

*Moringa oleifera* leaf is a rich source of minerals, such as calcium, potassium, zinc, magnesium, iron, phosphorus and copper [23], where it is represented in Table 1. One of the characteristics of the leaf is its high protein content, due to the essential amino acids, which constitute about 30% of its weight, being comparable to milk powder which contains 35%, and is available all year round, as the protein and essential amino acid content is present in the leaves, unlike other plants which contain them in the seeds [2,24,25], reporting a protein content of 29.4 g protein/100 g dry weight in the leaves. Thus, *Moringa oleifera* can be considered a new source of protein to be included in food, like chia seed, with a protein content of 24 g protein/100 g dry weight [26]. Regarding carbohydrates, its level is lower (8.1%) [27] compared to the other parts of the plant, as can be seen in Table 2. In addition, fibres were also found, with a value ranging between 18.1 and 21.1 g/100 g dry weight of the leaves [28].

The leaves are noted for high levels of β-carotene and provide more vitamin A than carrots and pumpkin [8], however it is not clear whether this vitamin is retained even after drying and grinding the plant. Even so, studies have shown that their consumption is sufficient to counteract the effects of this vitamin deficiency [2]. They are also a good source of B vitamins (quoted from the book tree miracle), among which thiamine, riboflavin and niacin have been found, with a concentration between 0.06 and 0.6 mg/100 g, 0.05 and 0.17 mg/100 g and 0.8 and 0.82 mg/100 g for thiamine, riboflavin and niacin, respectively. In the dried leaf, their concentrations were 2.85, 22.16 and 8.86 mg/100 g DW, respectively [29,30]. On the other hand, supplementation with 100 mg/dL of *Moringa oleifera* leaf per day has a similar effect to treatment with vitamin E at 50 mg/dL per day [8], contains more vitamin C than an orange and more calcium than dairy products, however a significant part of this calcium is present in the form of calcium oxalate crystals, which cannot be used by the body and is eliminated directly without being absorbed [2]. In addition, *Moringa oleifera* is high in potassium and iron; even more than bananas and spinach respectively [31].

*Moringa oleifera* seed has a high proportion of monounsaturated/saturated fatty acids (MUFA/SFA), sterols and tocopherols, as well as proteins rich in sulphur amino acids [32]. As reported in previous studies, *Moringa oleifera* seed oils (also called Behen oil, which is the commercial name given to *Moringa oleifera* oil) have similar fatty acid content and physicochemical parameters to those reported for other vegetable oils and can be considered as a healthy alternative to hydrogenated oils in food formulations. The main fatty acids present in *Moringa oleifera* oil are behenic, linoleic, stearic, palmitic, oleic, arachidic, linolenic, eicosenoic and heptadecanoic acids [25], with oleic acid being the main unsaturated fatty acid, with 73.5% in the seed oil [33]; carbohydrate content is 27.5% [27]. The seeds are a rich source of Ca and Mg, respectively [25,34].

Of the other edible parts of *Moringa oleifera*, with respect to carbohydrates, the pods contain 10.4%, stem 18.5%, bark 26.9% and stem with bark 31.1% [27]. Karuna et al. [35] found that the part with the highest level of fibre is the root (45.43%), compared to the stem (41.60%) and bark (25.73%). Immature pods and flowers are characterized by a higher content of total monounsaturated fatty acids (MUFA, 16–30%) and are low in PUFA (34–47%), compared to leaves [36]. The highest K content is found in vegetative parts and immature pods [25,34].

### 4.2. Secondary Metabolites

The different parts of *Moringa oleifera* are good sources of glucosinolates, flavonoids and phenolic acids [25,39], carotenoids [40], tocopherols [41]. Alkaloids, saponins, tannins, steroids, phenolic acids, alkaloids, carotenoids, polyphenols, isothiocyanates, phytates, glucosinolates, flavonoids and terpenes can be found in the *Moringa oleifera* leaf [7]. Among the glucosinolates, benzyl 4-O-(α-L-rhamnopyranosyloxy)-glucosinolate is the most predominant (glucomoringin) [25].

Its leaves include 11 phenolic acids (gallic acid, caffeic acid, chlorogenic acid, o-coumaric acid, p-coumaric acid, ellagic acid, gentisic acid, sinapic acid, syringic acid) [42,43] and their derivatives (coumaroylquinic acids and their isomers, feruloylquinic and caffeoylquinic), 26 flavonoids (present mainly as flavonol and glycoside: quercetin, rhamnetin, campferol, apigenin and myricetin [7]. Flavonoids include flavonol glycosides (glycosides, rutinosides and malonylglycosides) of quercetin “kaempferol” 0.05–0.67%) isorhamnetin and lignans (isolariciresinol, medioresinol, epipinoresinol glycosides and secoisolariciresinol) [37,38,39]. Furthermore, there is a difference according to geographical area, showing a higher phenolic content in Pakistan than in India, Thailand, Nicaragua and even in the United States [14,21]. The flavonoid composition is higher in the leaves than in the seeds, ranging from 2000 to 12,200 mg per dl of *Moringa oleifera* leaf.

*Moringa oleifera* seed contains phytosterols, the most abundant of which are β-sitosterol, stigmasterol and campesterol [3,44]. Alkaloids, saponins, phytates, tannin [45] and phenolic compounds (quercetin and p-hydroxybenzoic acid) [46] can also be found.

The seed is oleaginous and has aleurone sources with a lectin fraction, is an oil that must be refined to be consumed and contains a similar composition to oleic acid, however, the composition may vary according to the geographical location of the plant [3]. Seeds are a good source of glucosinalates (8–10%), glycosylate isothiocyanate, 4-(α-L-rhamnosyloxy) benzyl ITC [47] and 4-O-(α-L-rhamnopyranosyloxy)-benzylglucosinolate (glucomoringin) [25,47].

In other edible parts of *Moringa oleifera*, more than 102 bioactive compounds have been identified in the root, while 74 essential oils have been identified in the flowers. In addition, both the peel and the dried pod of *Moringa oleifera* fruit contain high levels of polysaccharides and glucans; 28% in the peel and 32% in the pod [48]. Glucosinolates have also been found, of which 4-O-(α-L-rhamnopyranosyloxy)-benzylglucosinolate (glucomoringin) is the most predominant in the stem, flowers and pods of *Moringa Oleifera* [25,47]. Although in the roots, benzyl glucosinolate (glucotropaeolin) is the most prominent [49], flavonoids, notably flavonol glycosides (glycosides, rutinosides, and malonylglycosides) of quercetin [kaempferol isorhamnetin] are also present in various parts of the plant, except in the roots and seeds [39].

Table 1 highlights the nutritional composition of the edible parts of *Moringa oleifera* and Table 2 highlights the nutritional composition of the *Moringa oleifera* leaf, since this plant organ is the most studied and most consumed [29], which concentrates most of the nutrients [8].

## 5. Biological Effects of *Moringa oleifera*

The bioactive compounds (Figure 1) present in *Moringa oleifera* confer properties associated with disease prevention and treatment, such as antimicrobial [50], anti-inflammatory [51], anticancer, antidiabetic, antioxidant, hepatoprotective and cardioprotective [52,53,54]. Primary and secondary metabolites may also be involved in these applications. Primary metabolites are proteins, polysaccharides and lipids involved in physiological functions. Among them, polysaccharides and fibres are the main compounds showing positive effects on chronic diseases such as cancer, cardiovascular diseases, diabetes and obesity. On the other hand, secondary metabolites are minor molecules, such as phenolic compounds, halogenated compounds, sterols, terpenes and small peptides. Most of the phytochemicals reported in *Moringa oleifera* offer potential in the prevention and treatment of diseases.

The anti-inflammatory effect is due to the content of flavonoids, alkaloids, tannins and glycosides, among which quercetin appears to inhibit NF-KB activation, producing an anti-inflammatory effect [55]. Other compounds with an anti-inflammatory effect include kaempferol derivatives, flavonol glycosides [56,57], aurantiamide acetate, 1,3-dibenzylurea [58,59], diterpenes, α- and β-amyrin [60], benzaldehyde 4-0-β-glucoside [56,57], β-sitosterol [43], rutin [61], and glucosinolate, mainly attributed to the glycosylate isothiocyanate, 4-(α-L-rhamnosyloxy) benzyl ITC, resulting from myrosinase [47]. *Moringa oleifera* reduces inflammation by suppressing inflammatory enzymes and proteins in the body, and leaf concentrate can significantly reduce inflammation in cells [62].

The antimicrobial effect provided essential oils from the leaves and alcoholic extracts of the seeds. In fact, Chuang et al. [48] demonstrated this activity of the leaf and leaves against dermatophytes such as *Trichophyton rubrum* and *Trichophyton mentagrophytes* [48]. In addition to these compounds, other compounds have been found that also produce this effect, 4(βL-rhamnosyloxy) benzyl isocyanate or pterigospermine,4-(β-D-glucopyranosyl-1→4-β-l-ramnopyranosyloxy),benzyl thiocarboxamide,(-)-Catechin, phenylmethanamine, 4β-D-glucopyranosyl-1-->4 β-L-rhamnopyranosyloxy)-benzyl isocyanate, niazirine [58,63,64] and glucosinolate mainly attributed to the glycosylate isothiocyanate, 4-(α-L-rhamnosyloxy) benzyl ITC, resulting from myrosinase [47].

Phenolic compounds have been associated with the antimicrobial and antifungal activities of *Moringa oleifera* extracts [65], the leaves being the organs with the highest amount of these compounds. Regarding the antimicrobial effect of *Moringa oleifera* plants when included in food, *Moringa Oleifera* contributes to control the growth of undesirable microorganisms, due to low pH values and the presence of pterigospermin [66]. The roots of *Moringa oleifera* have antibacterial properties and are described to be rich in antimicrobial agents. The bark extract has been found to have antifungal activities, while the juice of the bark and stem show an antibacterial effect against *Staphylococcus aureus* [67].

Studies have shown the anticarcinogenic effect of several compounds, namely glycosylated isothiocyanate, benzyl carbamate niazimycin and β-sitosterol, which have antitumour properties against lung, breast, skin, oesophageal and pancreatic cancer. These compounds are found in high concentrations in the leaves and seeds of the plant [68]. *Moringa oleifera* is rich in ascorbic acid, which provides an anti-diabetic effect by aiding insulin secretion, and another compound found in *Moringa Oleifera* that produces this effect is myricetin [69,70].

Antioxidants are popular because they scavenge free radicals that cause oxidative stress, cell damage and inflammation. *Moringa oleifera* contains antioxidants called flavonoids, polyphenols and ascorbic acid in the leaves, flowers and seeds [71]. Studies have shown that the plant is rich in polyphenols, which gives it a high antioxidant capacity. The compounds in *Moringa oleifera* that provide this activity are feluric, gallic and ellagic acids, β-sitosterol, myricetin, niazimycin, niacimicin A and B, tocopherols: α-tocopherol, δ-tocopherol, γ-tocopherol, vanillin, kaempferol, quercetin, β-carotene (-)-catechin, astragalin and isoquercetin [58,59,61,70,72,73,74,75,76,77].

*Moringa oleifera* plays an important role in protecting the liver from damage, oxidation and toxicity due to the high concentrations of polyphenols in its leaves and flowers. *Moringa oleifera* oil can also restore liver enzymes to normal levels, reducing oxidative stress and increasing protein content in the liver. The flowers and roots of the *Moringa oleifera* plant contain a compound called quercetin, which is known to protect the liver [78]. Other compounds contained in the plant with this activity are β-sitosterol, quercetin and some of its glycosides, rutin [61,75] and flavonoids, which also prevent lipid oxidation [79].

*Moringa oleifera* leaves and seeds have been found to help lower blood pressure; this is due to compounds called glycosides [78], and in the leaves it is also due to N-α-L-rhamnophyranosyl vincosamide [61]. *Moringa oleifera* leaf extract has also been found to significantly reduce cholesterol levels due to the action of β-sitosterol [78].

## 6. Toxic or Adverse Effects

*Moringa oleifera* is not entirely safe, as many studies have found various compounds that have been associated with major liver, kidney, haematological and other diseases. Roasted *Moringa oleifera* seeds contain potential mutagens such as 4-(α-lramnopyranosyloxy)-benzylglucosinolate, which increase the proportion of micronucleated polychromatophilic erythroblasts, indicative of some degree of genotoxicity [80]. The leaf has a high concentration of saponins, which can be potentially harmful for vegetarians, as their consumption reduces the bioavailability of divalent and trivalent metals such as Zn and Mg [81]. Moringin alkaloids, spirochin and the phytochemical benzothiocyanate have been found in the root and bark, toxic substances that predominate in the root and bark; the leaf was therefore identified as the safest edible part [11].

## 7. Applications of *Moringa oleifera* in Food Industry

*Moringa oleifera* has several uses due to its composition. The seed powder is used to purify water, eliminating a large amount of suspended material in rivers and turbid waters, making it a natural coagulant for water treatment. The oil from the seeds can be used as a fertiliser in plantations to encourage the growth of other species; it is also used for cosmetics such as soaps and perfumes [6], and even for the production of biodiesel [31]. *Moringa oleifera* extracts can be used to produce zeatin effective for plant development, increasing crop yields [82]. In addition to these applications, *Moringa oleifera* has been used in food, for example, in Mexico as an ingredient to partially replace fishmeal in tilapia feed, due to its protein and carbohydrate content [83].

## 8. Use of *Moringa oleifera* in Bakery Products

**Figure 2:**
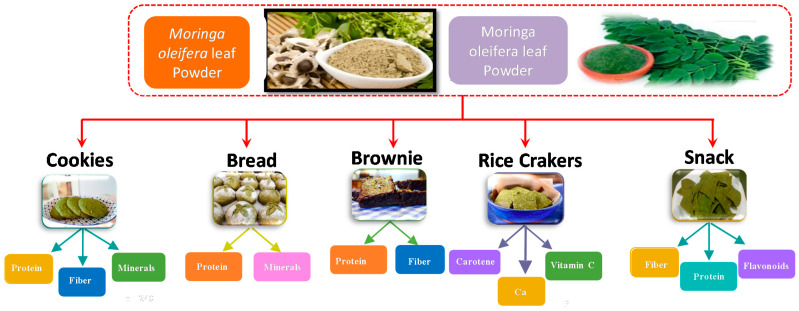
Applications of *Moringa oleifera* in bakery industry.

The use of *Moringa oleifera* in food can be very beneficial; some researchers indicate that food products can be enriched with *Moringa oleifera* by providing vitamins, minerals, essential amino acids and oils in order to improve their nutritional value [15] (Table 3 and Figure 2).

Supplementation of *Moringa oleifera* powder in cereal porridge has been shown to improve the nutritional value by increasing the vitamin A content by up to 15 times. In the white maize variety, the protein content by fortification increased by 94% with 15% *Moringa oleifera* powder and for the yellow maize variety, the protein content increased by 44% [24]. 

However, many studies have shown that products formulated with *Moringa oleifera* powder in high concentrations may be generally unacceptable to most consumers [24]. Domenech et al. [1] reported that most *Moringa oleifera* is used almost exclusively in breads, biscuits and meat products, with its nutritional, technological and preservative purposes, respectively [1]. 

*Moringa oleifera* supplementation has a nutritional purpose, although it can provide other benefits to the product, such as improved digestibility [15], dough stability, antioxidant capacity, preservation, among other benefits associated with the plant [1]. The use in bread and biscuits is a very useful strategy to make up for nutritional deficiencies, due to the high consumption of these foods. In addition, the food industry has tried to reduce the consumption of wheat flour in bakery products, in order to deliver foods that provide better nutritional characteristics, including reduced gluten, and for this, *Moringa oleifera* seems to be an option [84]. 

According to Ogunsina et al. [84], the incorporation of *Moringa oleifera* seed flour affects the organoleptic properties of different breads and biscuits; however, these differences are not significant when used in a ratio of 90% flour and 10% *Moringa oleifera* for bread and 80% flour and 20% *Moringa oleifera* for biscuits. Moreover, the taste was typical of *Moringa oleifera* seed, but acceptable in bread and the nutritional composition improved in both products, increasing the levels of protein, iron and calcium [84]. 

Chizoba et al. [85] also developed biscuits, substituting wheat flour with *Moringa oleifera* leaf flour; their results showed that the indicated proportion to maintain acceptable sensory characteristics and attributes is 90/10, i.e., to incorporate 10% of *Moringa oleifera* flour not exceeding 20% according to the authors [85].

The incorporation of *Moringa oleifera* seed meal in biscuits is reported to improve protein intake, increasing it by 45% to 90% if the addition of *Moringa oleifera* is 10% or 20% respectively. In the case of rice cakes, the addition of *Moringa oleifera* at 5% (freshly harvested) to 14% (dried) increases protein by approximately 26% [15]. 

Rabie et al. [86] also made biscuits fortified with *Moringa oleifera* leaf powder and seed powder in different concentrations, ranging from 2.5% to 7.5%. Their results found that supplementation with *Moringa oleifera* leaf powder has a higher amount of protein, ash, crude fibre, dietary fibre and minerals, while seed powder is characterised by a higher content of fat, protein, dietary fibre and minerals, and when mixed, a higher amount of essential amino acids was achieved, and when compared to the control, the biscuits with *Moringa oleifera* incorporation showed a lower carbohydrate content.

In relation to the physical characteristics, there is an increase in weight with a reduction in the volume and diameter of the biscuits, concluding that the best concentration to improve the nutritional characteristics without altering their organoleptic characteristics was 5% for the incorporation of leaf and seed powder and 2.5% + 5% in the case of mixing leaf and seed powder respectively [86].

Most of the evidence related to the incorporation of *Moringa oleifera* in cakes is associated with the consumption of biscuits, and in all reports, the results are similar. Supplementation increases nutritional value but affects physical characteristics, decreasing volume and colour in some instances; this was also demonstrated by a study by Nutan Narwal et al. [87].

The same would be true for the preparation of brownies and cakes with wheat flour, according to Santos et al. [88], who incorporated 5% and 10% *Moringa oleifera* leaf flour in chocolate brownies, indicating that the samples improved the nutritional value by increasing the ash content with a lower lipid contribution in relation to the control. A feature not mentioned in previous studies with biscuits is that the brownie showed the acidity of the product. The researchers concluded that there is no major difference between 5% and 10%, as similar results were obtained in both cases [88]. 

Whereas, in the wheat flour cake, the value of protein, moisture, crude fiber, total ash increased, with a reduction in lipids and carbohydrates, concluding that the cake sample with the addition of 4 g of *Moringa oleifera* was the most acceptable in terms of colour, flavour, aroma and overall acceptability [89]. 

In the case of bakery products, the use of *Moringa oleifera* powder in wheat flour bread dough as in other products increases the nutritional value, the protein and crude fiber content of wheat bread flour enriched with 5% *Moringa oleifera* powder has been found to increase from approximately 17% to 54% and 56% to 88% respectively. It should be noted that this improvement is accompanied by poor sensory properties, such as crust and crumb colour, as well as product weight and height [15]. 

Specifically for the case of whole wheat bread, El-Gammal et al. [90] added *Moringa oleifera* in different concentrations (5%, 10%, 15% and 20%); the results obtained indicated that *Moringa oleifera* leaf powder contained high amounts of protein and crude fibre, in addition to some essential minerals such as calcium, magnesium, phosphorus and iron. When *Moringa oleifera* was added to the preparation of wholemeal sliced bread, the protein content increased to 21.85%, the ash content (5.21%) and carbohydrate content decreased by 59.34%, and the intake of magnesium, calcium and iron increased compared to the control.

In relation to the undesirable effects of *Moringa oleifera* in bread incorporation, it negatively affects farinograph and extensometer values. Although there was an improvement in nutritional value, the acceptability of all loaf bread samples decreased with increasing levels of *Moringa oleifera* powder concentration, especially the loaf bread with 15 and 20%.

On the other hand, there are modifications in texture, taste, chewiness, elasticity of all samples compared to the control, however the researchers conclude that the best concentration to add to sliced bread is 5 or 10%, thus obtaining an increase in nutritional value with acceptable sensory characteristics [90]. 

Similar results were obtained by Bolarinwa et al. [91], who added *Moringa oleifera* seed powder to bakery products, increasing the protein value from 8.55 to 13.46%, ash from 0.63 to 1.76%, lipids from 7.31 to 15.75%, fibre from 0.08 to 0.62%, vitamin A from 50 to 74%, with a reduction in moisture from 22.9% to 20.01% and carbohydrates from 57.68% to 46.73%, also highlighting the increase in calcium, iron, phosphorus, and potassium in all its breads. Sensory evaluation results indicated that bread enriched with 5% *Moringa oleifera* seeds was not significantly different from the 100% wheat flour control [91].

Finally, while Devisetti et al. [92] evaluated the effect of *Moringa oleifera* leaf flour in sandwiches reaching similar conclusions, the protein content in puffed sandwiches increased, presenting 21.6 g in 100 g of product; while dietary fibre was presented at 14.8 g per 100 g of product, there was also a reduction in fat content of 3.7 g per 100 g of product with a high presence of phenolic compounds and flavonoids. In relation to the sensory characteristics of the sandwiches, an acceptable result was obtained in terms of texture [92].

## 9. New Trends 

New trends in the use of *Moringa oleifera* in bakery products focus on the preservation of its antioxidant power and on the use of dietary supplements in case of nutritional deficiency situations. In this context, according to the nutritional evaluation, it can be concluded that these components can be used as nutraceuticals [95,96,97,98]. The study of molecules of protein nature with bioactive effects on human health, such as antiproteolytic, procoagulant, antidiabetic, antihypertensive and anti-inflammatory properties, opens up new strategies for the design of nutritional supplements and the design of functional foods. This plant is also economical in all respects and has been used by many people due to its many advantages. Today, there is a great demand for plant-based medicine, food supplements, health products, pharmaceuticals, cosmetics, etc. in the national and global market. However, most of the quality of food supplements has not been well evaluated. In addition, there are some types of conventionally used foods, which have some ethnopharmacological importance, but have not been clinically tested. The main mission of this research is to develop the scientific rationale behind their use. This effort aims to explore the therapeutic uses of a food plant, which has medicinal importance in a wide range. The result shows that the plant provides a tremendous source of micronutrients and macronutrients. Therefore, it can be further explored for the development of nutritional supplements in the future, which may be useful to treat various diseases and thus promote a better quality of life.

On the other hand, current research has sought to replace wheat flour largely or entirely with *Moringa oleifera*, to obtain a gluten-free product that also has unique nutritional characteristics. Currently, the trend in the use of *Moringa oleifera* aims to improve the nutritional characteristics of fortified products, but also claims to be an excellent supplement for the treatment of various diseases, creating nutritious and nutraceutical foods [99].

## 10. Concluding Remark

Taking into account all the considerations outlined in this review, it is important to note that bioactive compounds from *Moringa oleifera* have a potential application in food, especially in bakery products. However, there are scarce results regarding the isolation of phytochemical compounds to identify new natural bioactive agents from *Moringa oleifera* plants and to better understand the role of these compounds in the food matrix. The study of these molecules is a starting point for a better understanding of the role in the food matrix and for a correct technological, nutritional and sensory development of functional foods.

In addition, the stability of nutrients and bioactive compounds in bakery products should be studied. Although several studies have reported on the functional properties of bioactive compounds, there are not enough studies on the digestibility and bioavailability of these bioactive compounds in both in vivo and in vitro systems. Therefore, more clinical trials should be conducted to demonstrate the functional properties of these bioactive compounds present in *Moringa oleifera*. 

Because it is important to consider that not all of the plant is 100% safe and that there are parts that can eventually harm people’s health. The most toxic part of *Moringa oleifera* is the bark and the root, and the safest part is the leaves; it is important to test their toxicity. In addition, sensory analysis is mandatory before developing the food matrix, because significant alterations in taste and flavour could occur if the level of *Moringa oleifera* is not adequate. Alterations in sensory characteristics can certainly be a barrier to food consumption.

## 11. Conclusions

This review aimed to highlight the bioactive compounds of *Moringa oleifera* plants and recent approaches to functional applications and the influence of these biocompounds on the functional characteristics of bakery products. The main food products based on *Moringa oleifera* plants were found to be high in dietary fibre and low in fat, suggesting that this plant can be used in the formulation of low-calorie food products.

The incorporation of *Moringa oleifera* will increase the nutritional value, improving the contribution of macro and micronutrients, of which proteins, fibres, vitamins and minerals are the most important, however, there is a difference when using the leaves versus the seed of *Moringa oleifera*, as the latter will increase the value of lipids, which is not a characteristic of the leaves. In all cases, high concentrations alter the physical and sensory characteristics of the supplemented products. In most studies, *Moringa oleifera* has been used in bread, biscuits and brownies, giving us clues to follow in bakery or bakery products due to their similarity in ingredients and types of preparations. It is possible to develop food products based on *Moringa Oleifera* flour with acceptable sensory and nutritional properties when less than 20 g of this material is used, depending on the intended food product.

Most of the studies are on biscuits, however, the addition of *Moringa oleifera* can be found in meats, juices and yoghurts among others. The addition of *Moringa oleifera* in the food industry represents a great contribution to the nutritional value of products, which can be an interesting strategy that aims to improve the nutritional status and health of people, especially in places of extreme poverty or food scarcity. Moreover, it is an attractive factor for those who like to eat healthy and that a food product is more than just taste and meaning. 

In conclusion, taking into account all aspects related to the level of plant utilization, this review reported that *Moringa oleifera* can be used in the production of nutrient-enriched bakery products with good storage quality and high consumer acceptance.

## Figures and Tables

**Table 1:** The nutritional composition of the leaf of *Moringa oleifera*.

Minerals (mg)	Fatty Acids %	AA Essentials (mg)	Vitamin	Bioactive Molecules (mg)
P 112.1	C16:0 23.3	His 700–1357	Vitamin A 11,300–23,000 UI	β-carotene 6.6–17.6 mg
Mg 10.6	C16:1 0.4	Thr 790–2197	Vitamin C 18.7–140 mg	α-Tocopherol 74.5–122.1
Na 224.1	C18:0 4.1	Tyr 480–1880	Riboflavin 22.6 mg	Thiamine 2.85 mg
K 2071.9	C18:1 6.27	Val 1130–2758	Niacin 8.86 mg	Polypherols 2.10 –12.2 mgGAE/mg
Mn 8.37	C18:2 6.11	Met + Cys 140–835		Flavonoids 5.1–12.2 mg/g
Cu 0.95	C18:3 56.9	Leu 1750–4289		Myricetin 5.8 mg/g
S 137	C20:0 0.21	Phe 890–2714		Quercetin 0.21–7.6 mg/g
Cr < 0.5	C22:0 0.70	Lys 1325–1530		Kaempferol 4.6 mg/g
Mb 0.75				Gallic Acid 1.03–1.34 mg
Ni < 0.5				Chlorogenic Acid 1.8–6.97 mg/g
Se 2.71				Glucosinalates 21.84–59.50 mg/g
				Tannins 132–1200 mg
				Oxalates 430–1600 mg
				Phytates 250–2100 mg

Source José J., García L. [16]; Castro-López et al. [37]; Rodríguez-Pérez et al. [38].

**Table 2:** Nutritional composition of *Moringa oleifera* edible parts.

Component/100 g Dry Weight	Leaves	Immature Fruit Pericarp	Seeds
*Macronutrients*			
Energy (kcal)	205–295.6	178.2	564.5
Protein (%)	19–27.1	17.2–19.3	32.9–38.3
Lipids (%)	4.7–5	0.4–1.3	30.8–44.8
Fiber (%)	7.9–19.2	22.6–46.8	4.9–15.9
Carbohydrates (%)	27–51.7	21–51	14.4–16
*Minerals*			
Calcium (mg)	1.875–2.076	12.5–29	76.9
Iron (mg)	27.8–38	2.3–5.3	13.7
*Fatty Acids*			
C18:1 Olecic Acid	6.27	18	67.9–78
*Others*:			
Ascorbic Acid (vit c) (mg)	18.7–140	871	84.5
Chlorophyll (mg)	126.8	-	-

Source José J., García L. [16]; Castro-López et al. [37]; Rodríguez-Pérez et al. [38].

**Table 3:** Results on the applicability of *Moringa oleifera* in bakery products and its effect on product quality.

Bakery Products	Parts Used	*Moringa oleifera* Application/Concentration Used	Main Results/Conclusions	Reference
Cookies	*Moringa oleifera* leaves and seeds; and a combination of both.	2.5%, 5.0%, 7.5%	*Moringa oleifera* raised the nutritional value highlighting the amount of protein, ash, fibre and minerals. In addition, it showed an increase in weight without increasing the volume of the cookies compared to the control.	[2]
Bread and cookies	*Moringa oleifera* seed flour (MSF).	10%, 20% 30%	Bread with 10% and cookies with 20% of MSF respectively had more protein, iron and calcium.	[23]
Cookies	Leaf flour (LF)	10%,20%,30%,50%	Incorporation at 10% of LF showed better sensory attributes; however, acceptability decreased as *Moringa oleifera* levels increased.	[26]
Cookies	Leaf flour (LF)	5%	*Moringa oleifera* supplementation at 5% of LF raised the nutritional value of proteins and ash, showing a lower content of carbohydrates.	[48]
Cookies	Leaf flour (LF)	0%,10%,20%,30%,50%	The best acceptability in wheat flour biscuits supplemented with *Moringa oleifera* was shown by the concentration at 10% of LF	[85,93]
Brownie (cake)	Leaf powder (LP)	0%, 5%, 10%	It improved the physicochemical characteristics; and a higher ash content and lower lipid content was found, compared to the control sample.	[37]
Bread	Leaf powder (LP)	5%, 10%, 15%,20%	Supplementation of LP in bread raised the nutritional characteristics of proteins, ash and minerals; however, the carbohydrate content decreased. The acceptability decreases as the incorporation of *Moringa oleifera* increases, so the greater acceptability is at 5% and 10% of LP.	[79]
Bread	Seed powder (SP)	0–20%	The results showed an increase in the value of proteins, minerals, ash, lipids, and fibre; however, there was a decrease in the value of carbohydrates. There were no sensory differences with the control when incorporating 5% of SF.	[65]
Snack	Leaf powder (LP)	20%	Snacks increased their nutritional value in protein and fibre, showing a low amount of fat. Furthermore, flavonoids were found in the final product.	[66]
Rice crackers	Moringa leaves (ML)	1%, 2% y 5%	1% and 2% of ML had higher levels of β-carotene, vitamin C, and calcium than the control. Sensory scores were comparable to control even at the end of the storage test.	[94]
Bread	Moringa leaf powder (MLP)	0%, 1%, 2%, 3%, 4% y 5%	The nutritional composition of proteins, ash, fibre, minerals and β-carotene improved. Acceptability decreased when *Moringa oleifera* supplementation increased, affecting the bread physical and sensory attributes.	[93]
